# A case of facial nerve palsy in a pediatric patient associated with Covid-19

**DOI:** 10.1186/s13052-022-01263-0

**Published:** 2022-05-16

**Authors:** Alessandra Iacono, Elisa Pennisi, Consuelo Benincasa, Federico Marchetti

**Affiliations:** 1 Department of Pediatrics, Santa Maria delle Croci Hospital, Postal address: 5 Vincenzo Randi Street, 48121 Ravenna, Italy; 2 Department of Medical Sciences, Pediatrics, University of Ferrara, Ferrara, Italy

**Keywords:** Facial nerve palsy, COVID-19 infection, Neurologic manifestation

## Abstract

**Background:**

Pediatric facial nerve palsy is acute and mostly idiopathic; other causes are post-infectious forms.

**Case presentation:**

We describe a rare case of facial nerve palsy associated with COVID-19 in a 5-year-old boy. The diagnosis of post-infectious COVID-19-related facial paralysis was made by serology positivity for a previous infection (IgG positive, IgM and IgA weakly positive), in the presence of a negative molecular nasopharyngeal swab and in the absence of other etiologies. Early treatment with steroids (1 mg/day for 7 days followed by tapering) and supportive care solved the problem.

**Conclusion:**

In a child with facial paralysis, COVID-19 must be considered as the cause and both nasopharyngeal swab and serology must be performed.

## Background

Facial nerve palsy is an acute-onset, unilateral paralysis of the facial musculature caused by the isolated dysfunction of the peripheral facial nerve [1, 2]. The most common cause is idiopathic (Bell’s palsy), responsible for 60–80% of cases. Other relevant etiologies include post-infectious complications (upper respiratory tract infections such as otitis, herpetic viral infections, neuroborreliosis) [3].

According to recent studies in literature, an unusual increase in children with COVID19-related facial palsy was observed in the period from March 2020 to April 2020 [3]. The coronaviruses have an affinity for angiotensin converting enzyme 2 (ACE2) receptors normally located on the human epithelial cells of the respiratory tract but also on the central nervous system, which explains the neurological symptoms reported in many patients.

Here, we present a case of facial nerve palsy presenting in a 5-year-old boy. We aimed to provide another rare pediatric case, to better understand the clinical features, pathogenesis and prognosis.

## Case presentation

A healthy 5-year-old boy presented with right facial paresis (incomplete closure of the right eye and deviation of the buccal rim) that appeared approximately 10 days prior to admission. Concurrently, the mother reported a “change in mood”. The symptoms were accentuated when the child cried, and no fever was reported in previous weeks. Additionally, the mother did not report any tick bites.

No neurological disorders or neuropsychiatric disturbances were previously reported in his medical history nor in his family.

**Fig. 1:**
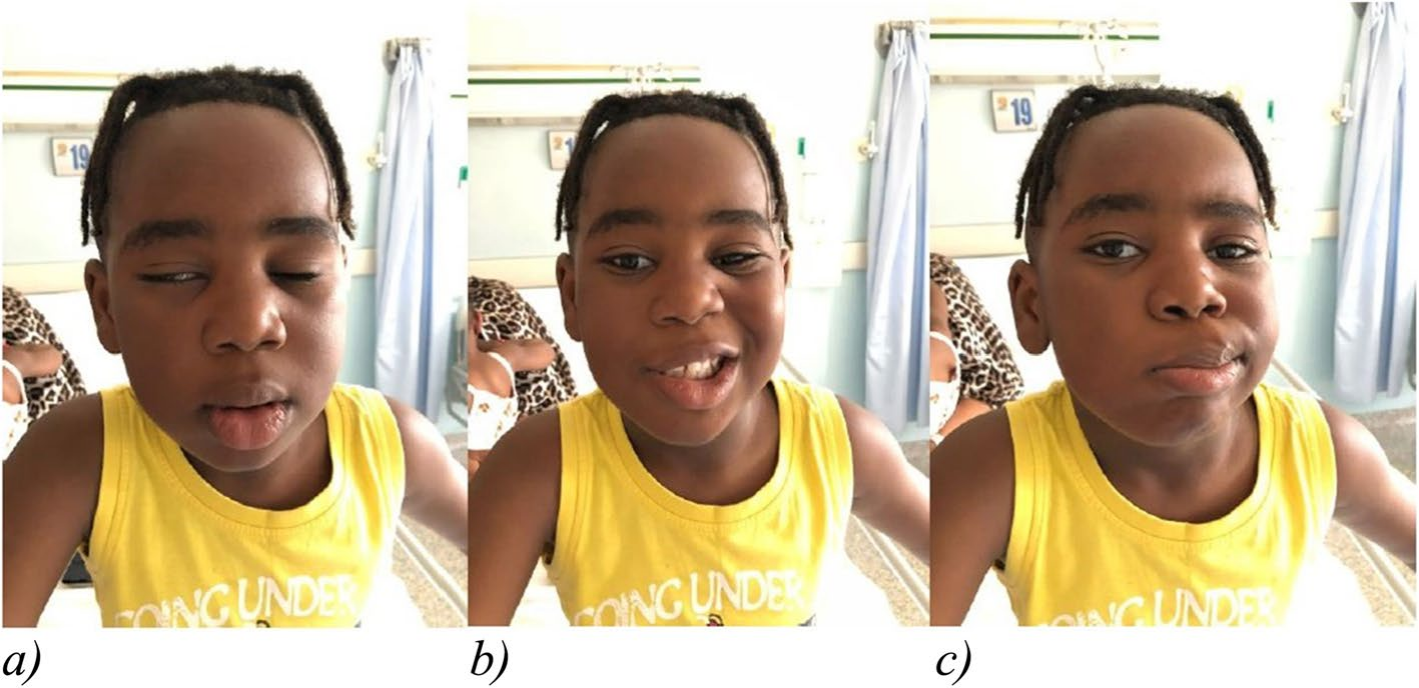
Right facial paresis. Legends: The child shows incomplete closure of the right eye (**a**), buccal border deviated to the left (paretic right labial angle) (**b**, **c**)

Upon physical examination, the child was in good general conditions, showing incomplete right eye closure, buccal rim deviated to the left (right labial angle paretic) (Fig. 1), no nystagmus. No additional clinically relevant findings were observed during the physical examination. Specifically, the tympanic membranes presented no acute inflammation.

A diagnosis of facial palsy with a Grade IV from the House-Brackmann grading system (obvious facial weakness, incomplete eye closure, no forehead movement, asymmetrical mouth movement, and synkinesis) was made.

Laboratory data showed normal blood count, negative indices of inflammation (CRP 0.5 mg/L, ESR 2), and normal coagulation. The only significant finding was an exceeding D-dimer of 4263 μg/L, (normal values < 500). The D-dimer values were still increasing the next day (15,353 μg/L).

The brain MRI (Magnetic Resonance Imaging), performed due to the alteration in the behavior, did not show any cerebral lesions or ischemic events. The study of the supra-aortic trunks with echo color Doppler showed no signs of steno-obstructive lesions in the common carotids, internal carotids and external carotids, with well-modulated flow in all carotid trunks; the optic fundus was normal.

Lymphoproliferative disease was excluded by means of light microscope examination of peripheral blood, normal lactate dehydrogenase (LDH) and normal abdominal ultrasound. In light of the possible post-infectious etiology of facial paralysis, the main serology tests were performed (EBV as previous infection, Parvovirus negative, HSV 1/2 IgG positive, HSV 2 IgG negative, Mycoplasma, Enterovirus, Borrelia, Bartonella negative).

Serology for SARS-CoV-2 revealed previous infection (IgG positive, IgM and IgA weakly positive) although the nasopharyngeal polymerase chain reaction was negative.

Given the positive of serology for SARS-CoV-2, although in absence of high indices of inflammation and lack of diagnostic criteria for multisystem inflammatory syndrome in children (MIS-C), an echocardiogram and electrocardiogram were performed and resulted as normal.

The diagnosis of post-infectious COVID-19-related facial paralysis was made and steroid therapy with prednisone (1 mg/day for 7 days followed by tapering) and eye lubricants was immediately started. At 1 month follow-up, the child was healthy and was no longer showing any sign of palsy.

## Discussion

Facial nerve palsy is an acute-onset, unilateral paralysis of the facial musculature caused by the isolated dysfunction of the peripheral facial nerve [1, 2]. The condition occurs more frequently in adults but is also seen in pediatric patients [1, 4]. Its incidence is 5–21/100000 children per year [3, 5].

In our case, only one finding of previous HSV infection (anti HSV 1/2 IgG positive, anti HSV 2 IgG negative) was found, which is particularly frequent in the general and pediatric population. For this reason, associated with the complete absence of clinical signs and symptoms of HSV on physical examination and in the immediate history (i.e. no active HSV or herpes zoster lesions were found in the oral mucosa, no lymphadenopathy was observed), HSV was not considered as a likely etiological cause of facial paralysis. Similarly, EBV, Parvovirus, Mycoplasma, Enterovirus, Borrelia and Bartonella were also excluded.

Facial nerve palsy is normally self-limiting, and symptoms commonly resolve within weeks to months. However, in a few cases, it can result in longer-term facial muscle weakness or in sequelae such as lagophthalmos, exposure keratopathy syndrome, ocular dryness and swallowing dysfunction [1].

According to recent studies in literature, incidence of COVID-19—related facial palsy increased during the pandemic both in adult and pediatric emergency departments. A neuroinvasive tropism of the SARS-CoV-2 virus family has already been reported, suggesting a similar potential for COVID-19 and raising an alarm about possible neurological involvement in these patients [3].

COVID-19 related complications such as acute necrotizing encephalopathy, hypoxic encephalopathy, ADEM, severe encephalopathy with white matter and corpus callosum lesions, and acute fulminant cerebral edema have already been reported [6, 7]. In milder COVID-19 cases, peripheral nervous system manifestations are predominant, including not only dysgeusia and anosmia, but also Guillain-Barré, Cranial Polyneuritis, and Miller-Fisher syndrome [8]. Another event post SARS-CoV-2 infection is pediatric acute onset neuropsychiatric syndrome (PANS) which presents with a sudden onset of obsessive-compulsive disorder (OCD) or a severely restricted food intake, and concurrent neuropsychiatric symptoms and motor dysfunction [9].

COVID-19-related pediatric cases appear to have a milder course of disease. Nevertheless, an increasing number of cases of acute idiopathic paralysis of the facial nerve has been reported, which are usually the first symptoms to appear or occur during the acute phase within the first week of viral symptoms onset [10]. However, it is important to take this manifestation into consideration also a few weeks after the acute phase, when serology is positive and the nasopharyngeal swab is negative.

The possible pathogenesis includes formation of microthrombi with ischemia of the vasa nervorum and demyelination induced by the inflammatory process so as to determine an increase in D-dimer [11, 12]. The increase of D-dimer is non-specific, and this is a signal of an acute infectious event. In this case, however, considering the negativity of the other infectious agents and the presence of COVID-19 antibodies, this association can be predicted. Therefore, laboratory data cannot be considered as a casual finding not related to a SARS-CoV-2 infection, since we attest that Bell’s palsy is one of the manifestations of COVID-19. Lastly, the mood change reported by the mother is not described in patients with Bell’s palsy, however it is described in patients with a previous SARS-CoV-2 infection.

In addition to vascular damage, another mechanism could be the functional alteration of the ACE2 receptor, which follows the binding of SARS-CoV-2 with ACE2 expressed in various regions of the nervous system, which causes a cytokine storm and therefore inflammatory response.

Furthermore, infection causes a significant reduction in the number of CD4 + T cells, CD8 + T cells, B cells, NK together with lymphocytes, monocytes and eosinophils, with immunosuppression and increased susceptibility to infection and reactivation of latent infections [6]. Finally, the direct binding of COVID-19 spike with glycolipids of the superficial peripheral nerves causes damage [13].

Studies among the adult population highlight that SARS-CoV-2 IgM can be found in blood samples 5 days after the onset of COVID-19 symptoms, persisting for at least month with a subsequent gradual decrease [14]. Otherwise, the mean duration of SARS-CoV-2 IgG antibodies detection is 14 days. Antibodies (SARS-CoV-2 IgG + IgM) can also be positive in patients who have had no common COVID-19 symptoms such as fever, cough, sore throat and respiratory difficulties. This evidence is in line with the absence of other signs and symptoms except for the facial palsy in our patient and with SARS-CoV-2 IgM test mild positivity and IgG test positivity during our evaluation which took place 10 days after the presentation of the palsy.

The treatment includes steroids and supportive care (eye lubricant); antiviral therapy has shown effective results in the acute management of adults and in one pediatric case [10, 11, 15].

Literature has reported 8 children with peripheral facial nerve palsy associated with COVID-19- All children received oral steroid therapy. In one case, intravenous acyclovir was administered as the child had hyper IgM syndrome and was in the acute phase of the disease (nasopharyngeal swab positive) [1, 3, 10, 15].

COVID-19-related facial nerve paralysis appears to have a positive outcome in children, nevertheless large scale studies are still required to confirm this finding.

## Conclusions

This is a rare case of facial nerve palsy associated with COVID-19, presenting in a 5-year-old boy. Current state of research reveals only a few of such cases in pediatric age. Therefore, findings showed that in the case of a child with paralysis of the facial nerve, it is necessary to evaluate a nasopharyngeal swab as well as serology for COVID-19, since the neurological manifestation can still occur weeks after the infection. Although not enough to establish a clear causative relationship between the two conditions, these data may suggest that COVID-19 could also manifest itself as facial paralysis alone.

## Data Availability

All data generated during this case report are included in this published article.

## Abbreviations

ACE2: angiotensin converting enzyme 2
ESR: erythrocyte sedimentation rate
CRP: C-reactive protein
MRI: Magnetic Resonance Imaging
LDH: lactate dehydrogenase
MIS-C: Multisystem inflammatory syndrome in children
PANS: pediatric acute onset neuropsychiatric syndrome
OCD: obsessive-compulsive disorder
